# Supplementary material for: Egg masses as training aids for spotted lanternfly Lycorma delicatula detection dogs
Source: PLoS One. 2021 May 3;16(5):e0250945. doi: 10.1371/journal.pone.0250945 (PMC8092771; doi:10.1371/journal.pone.0250945)
Supplement: Attachment — Submitted filename: SLF_MS_ResponseToReviewers.docx [file pone.0250945.s002.docx]

Reviewer #1: 02/04/2021


Spotted lanternfly (SLF) is an exotic pest of various agricultural and forest crops in North America. The current research deals with the potential of dogs in the detection of the egg masses. It’s an exciting small study that could offer a new tool for the fast and reliable survey of this pest. It should be of interest to this journal. However, the entire manuscript suffered from the lack of discipline in writing. Mistakes in the description of SLF biology will also need to be corrected before it can be considered for publication.


Major concerns:

1. Write scientifically by using clear and concise sentences. Stay focus on the subject matter for each paragraph. Exclude unrelated information. Don’t mix up information from different subjects, e.g., information belongs to Material and Methods should not be included in Results, etc. I have made some suggestions below, but more thorough editing is needed to improve the manuscript.

*Thank you, the manuscript has been edited for clarity.*

2. Understanding none of the authors were trained in entomology, critical review of the manuscript from a qualified entomologist is strongly recommended to ensure proper use of nomenclature and correct description of other facts about this pest.

*We have spoken with Dr. Julie Urban, who works with SLF extensively, and have made comments based off of her reading of the manuscript as well. She is also acknowledged the acknowledgments.*

3. The introduction portion of SLF biology (ln 55-72) need a complete overhaul, with the first paragraph focus on hosts and damages, and the second paragraph on life cycle and egg stage.

4. SLF egg masses are mostly found on tree trunks and upper branches, with a small proportion on stones and other nonliving substrates on the ground. I do not see canines being actively used to detect egg masses in real field situations. Maybe more useful in cargo and commodity inspections as part of the quarantine enforcement?

*It is true that since the drafting of this MS and the introduction of an SLF detection canine by PA Dept of Ag, implementation of this work has been utilized in cargo and commodity inspections as well as nurseries. We have modified the manuscript to better capture that.*

5. How do you match the controls? Ideally, you should match target odor with control odor from the same tree species for both dead and live egg masses. Was that how you set up the trials? More explanation is needed.

*In general, with the training sets, we did not always have the knowledge of the exact tree that a mass was from. We did include controls of the same species of tree. In particular with the double-blind testing, the controls were from the same exact tree as the egg mass. This is now more clearly expressed in the manuscript.*

Specific comments:

1. No need to credit photos taken by one of the co-authors.
*Photo credit has been removed.*

2. ln 1. Should be “Egg mass” not “Egg Masses”.
*Reworded title.*

3. ln 33-34. Awkward writing. Rephrase please.
*Modified writing.*

4. ln 56. “Vietnam”. Not true anymore. Delete.
*Thank you – removed.*

5. ln 57. “eating the phloem”. Not true. SLF is a sap feeder. Also, there is no need to include the definition of phloem.
*Given our discussion with Dr. Urban we have modified this section and the information on the SLF generally.*

6. ln 58-60. Awkward writing. Rephrase please.
*Modified.*

7. ln 65. Confusing.
*Modified.*

8. ln 84-86. This does not belong here. Move to the last paragraph n introduction as part of the study objectives.
*This sentence is about the Wallner and Ellis study from the prior sentence and has been modified to be clearer.*

9. ln 109-111. This should be included in the “Ethics Statement” with a few more words on the use of the canines.
*We have added an “Ethics Statement” section within the Methods section of the manuscript.*

10. ln 196-197. Citation needed.
*We have updated this sentence and added a citation.*

11. ln 284-286. Delete. Already described in M&M.
*Deleted.*

12. ln 310-314. These equations belong to M&M, not here.
*Moved.*

13. ln 359. “immediately spontaneously”. Awkward wording. Re-word.
*Removed “immediately”.*

14. ln 362-365. Good point.

15. ln 373-375. Would be interesting to see what’s responsible for the scent extract.

Reviewer #2: In this study, the authors present detection dogs as a method to find Lycorma delicatula egg mass. Throughout the manuscript, the methodology of training detection dog is presented and analyzed well. Nevertheless, the manuscript contains two major issues to be resolved before the article is to be published: an in-depth consideration of the target organism itself and detection efficacy of the method in the field.

First, although the paper focuses on the training method of detection dogs, it does not have deep enough consideration on the target organism itself. Although the paper describes general phenology of the insect, we were unable to find information on the oviposition behavior or characteristics of egg mass itself, which is the target of detection. For example, what is the major oviposition host plant of L. delicatula, or at which height or substrate are the egg masses often be found? Without the considering the biology of L. delicatula and its egg mass, we may not be able to apply the method in the field.
*We agree with this comment, and have reframed the introduction to describe search scenarios that dogs are more likely to be utilized for, including nurseries, cargo, and automobiles, rather than trees specifically where, it is true, many of the egg masses are far too high up for the dogs to (likely) be especially effective.*

Second, there is no field testing of the method presented in the study. As L. delicatula lays eggs on a variety of substrates including metal fence or host plants, the efficacy of the method may vary on the environment. Furthermore, recent study suggests that L. delicatula eggs can be found on tree-of-heaven or black walnut trees from up to 18 m above the ground (Liu and Hartlieb 2020). Especially, most favored egg heights were 8-10 m for tree-of-heaven and 4-6 m for black walnut. In this case, can dogs detect eggs at this height, or even if it does, is there a way to confirm its presence? The paper at least should address a more in-depth concerns on lack of efficacy experiment.
*We agree with this and like above, it is unlikely that dogs used for this will be searching trees, but rather cargo, rail and cars, among other lower items. We have included in the introduction more specifics about the likely implementation of these dogs, once trained.*

The following contains comments on the manuscript:
Manuscript
1) L65 – 72: Although the paragraph describes oviposition of L. delicatula, it lacks a more in-depth information on egg mass, which is the target of detection. For example, what is the most preferred host plant/ substrate? How big is an egg mass? Is there just one egg mass per tree or can more than one egg mass be found? What are the characteristics of the egg mass substrate (height/ surface texture etc.)
*We have modified and added to the introduction significantly, including these suggestions.*

2) L407 – 412: It describes that further research is required to test the efficacy of the detection method. Nevertheless, we would appreciate if this can be described in more details. For example, how confident are you that the method will work perfectly outdoor as well? Where would you find L. delicatula egg masses and what potential factors may limit the application of the methods? Also, if you find them, what is to be done for control of the egg masses? Limitation of the manuscript needs to be discussed in more detail.
*We have modified the discussion to include more appropriately the likely use of detection dogs in this arena, and how the data presented here may help future spotted lanternfly detection dogs.*

3) Few editorial corrections are to be made in references – scientific names of insects need to be italicized
*References have been modified.*
