# Supplementary material for: Egg masses as training aids for spotted lanternfly Lycorma delicatula detection dogs
Source: PLoS One. 2021 May 3;16(5):e0250945. doi: 10.1371/journal.pone.0250945 (PMC8092771; doi:10.1371/journal.pone.0250945)
Supplement: Attachment — Submitted filename: Review_PlosOne.docx [file pone.0250945.s003.docx]

Overall, the authors revised the manuscript according to the comments made by the reviewers. Once the following minor points are addressed by author, the manuscript is ready to be published.

First, this is a minor recommendation that may improve the manuscript. Because the paper focuses on detection of *L. delicatula* egg mass on artificial substrates including cargo, rail, and automobiles it may strengthen the importance of screening for these substrates by highlighting the potential spread of *L. delicatula* via these transportation means. This can be done by 1) citing different invasive insects that rapidly expanded its geographical range by hijacking trains and automobiles, 2) citing previous studies that observed potential *L. delicatula* dispersal via train (Flight Dispersal Capabilities of Female Spotted Lanternflies (Lycorma delicatula) Related to Size and Mating Status, Wolfin et al. 2019).

Second, the authors must describe the order and family of an insect when first mentioning its scientific name for all the insects. E.g. *Lycorma delicatula* (Hemipera: Fulgoridae). Also, if scientific name of an organism has already been mentioned, abbreviate its generic name in second time. E.g. *Ailanthus altissima ->* *A. altissima.*

Finally, the following comments address some minor editorial changes to be made and confusing sentences that need to be rephrased.

1. Ln 55: missing a period
2. Ln 68: space
3. Ln 72 – 75: confusing. Rephrase.
4. Ln 82 – 84: rephrase
5. Ln 101 – 104: add references
6. Ln 107 – 109: rephrase
7. Ln 360 – 362: rephrase
