# Supplementary material for: Egg masses as training aids for spotted lanternfly Lycorma delicatula detection dogs
Source: PLoS One. 2021 May 3;16(5):e0250945. doi: 10.1371/journal.pone.0250945 (PMC8092771; doi:10.1371/journal.pone.0250945)
Supplement: Attachment — Submitted filename: SLF_PLOSONE_ResponsetoReviewers2.docx [file pone.0250945.s004.docx]

Reviewer #1

All previous concerns have been properly addressed by the authors. Therefore, I recommend its publication by the journal with a few minor changes below:

1. At the first citation of a genus/species, authority, order, and family should be given in parentheses.

*We have added (Order: Family) for each insect and tree species noted in the manuscript, on their first citation.*

2. Be consistent when using the insect or plant names, e.g., don’t not use “The spotted lanternfly” in one sentence, and use “SLF” in another.

*We have replaced “SLF” in the manuscript with spotted lanternfly.*

3. ln 33. Replace “The spotted lanternfly” with “It” since it was used in the previous sentence.

*Replaced.*

4. ln 192-193. I believe the correct common name for Prunus serrullata is Japanese flowering cherry.

*Thank you – replaced.*

5. ln 271, 274, 277, etc. acronym “SLF” was not defined and therefore should not be used.

*The acronym “SLF” was removed throughout the manuscript and replaced.*

Reviewer # 2

Overall, the authors revised the manuscript according to the comments made by the reviewers. Once the following minor points are addressed by author, the manuscript is ready to be published.

First, this is a minor recommendation that may improve the manuscript. Because the paper focuses on detection of *L. delicatula* egg mass on artificial substrates including cargo, rail, and automobiles it may strengthen the importance of screening for these substrates by highlighting the potential spread of *L. delicatula* via these transportation means. This can be done by 1) citing different invasive insects that rapidly expanded its geographical range by hijacking trains and automobiles, 2) citing previous studies that observed potential *L. delicatula* dispersal via train (Flight Dispersal Capabilities of Female Spotted Lanternflies (Lycorma delicatula) Related to Size and Mating Status, Wolfin et al. 2019).

*Specific highlight of this species (and invasive insects in general) using railways as a means of transport has been added to the introduction, thank you.*

Second, the authors must describe the order and family of an insect when first mentioning its scientific name for all the insects. E.g. *Lycorma delicatula* (Hemipera: Fulgoridae). Also, if scientific name of an organism has already been mentioned, abbreviate its generic name in second time. E.g. *Ailanthus altissima ->* *A. altissima.*

*Thank you – the order and family for the spotted lanternfly were added, and scientific names were abbreviated where necessary.*

Finally, the following comments address some minor editorial changes to be made and confusing sentences that need to be rephrased.

1. Ln 55: missing a period
2. Ln 68: space
3. Ln 72 – 75: confusing. Rephrase.
4. Ln 82 – 84: rephrase
5. Ln 101 – 104: add references
6. Ln 107 – 109: rephrase
7. Ln 360 – 362: rephrase

*Thank you – all of the above have been modified.*
